# Synthesis, Characterization, and Sensitivity of a CL-20/PNCB Spherical Composite for Security

**DOI:** 10.3390/ma11071130

**Published:** 2018-07-03

**Authors:** Yanfang Zhu, Yuewen Lu, Bing Gao, Dunju Wang, Changping Guo, Guangcheng Yang

**Affiliations:** 1 Sichuan Co-Innovation Center for New energetic materials, Southwest University of Science and Technology (SWUST), Mianyang 621010, China; zhuyanfang806@126.com (Y.Z.); 18784030734@163.com (Y.L.); 321gaobing@163.com (B.G.); wangdunju@swust.edu.cn (D.W.); 2 Institute of Chemical Materials, China Academy of Engineering Physics (CAEP), Mianyang 621900, China; ygcheng@caep.cn

**Keywords:** CL-20, PNCB, micro-scale spherical composite, ultrasound-assisted emulsion method

## Abstract

Highly energetic materials have received significant attention, particularly 2,4,6,8,10,12-hexanitro-2,4,6,8,10,12-hexaazaisowurtzitane (CL-20). However, the application of this material was limited due to its high sensitivity. It is well known that the shape, size, and structure of energetic materials (EMs) significantly influence their sensitivity. At present, there are several ways to reduce the sensitivity of CL-20, such as spheroidization, ultrafine processing, and composite technology. However, only one or two of the abovementioned methods have been reported in the literature, and the obtained sensitivity effect was unsatisfactory. Thus, we tried to further reduce the sensitivity of CL-20 by combining the above three methods. The as-prepared composite was precipitated from the interface between two solutions of water and ethyl acetate, and the composite was insensitive compared with other reported CL-20-based EMs. The H_50_ value for the composite ranged up to 63 cm. This approach opens new prospects for greatly reducing the sensitivity of high Ems.

## 1. Introduction

Energetic materials (EMs), a class of materials with a large amount of stored chemical energy, are widely used in military and civilian applications [1,2,3]. Considering security, these materials have been researched extensively to gain insights into safety during transportation and storage [4,5]. It is well known that the shape, size, and structure of EMs significantly influence the sensitivity, thermal decomposition, and packing density [6].

To the best of our knowledge, various methods have been reported to decrease the sensitivity of EMs, including spheroidization, ultrafine processing, and composite technology [7,8,9,10,11,12]. One should note that the spherical crystals of EMs exhibit improved performance compared to non-spherical crystals, particularly regarding insensitivity, large packing density, and flowability [7,8,9]. Meanwhile, EMs with microparticle or nanoparticle sizes show insensitivity compared larger particles [10]. Therefore, some scientists are concentrating their attention on the production of micro/nano sized monodisperse spherical EMs [13]. However, compared to inorganic EMs, organic EMs are more difficult to prepare with the micro/nano spherical structure, because most organic energetic compounds exhibit van der Waals forces or other weak intermolecular interactions among molecules [14,15]. Apart from the above-mentioned methods, another method to reduce sensitivity is to use composite technology [11,12]. Based on previous research results, Yang et al. [11] reported the preparation and properties of core-shell CL-20/TATB composites. Wang et al. [12] studied a novel cocrystal explosive of 2,4,6,8,10,12-hexanitro-2,4,6,8,10,12-hexaazaisowurtzitane (HNIW) with desirable properties. However, the obtained H_50_ value was unsatisfactory.

2,4,6,8,10,12-hexanitro-2,4,6,8,10,12-hexaazaisowurtzitane (CL-20) is one of the most promising high energetic materials because it exhibits high density and large formation enthalpy [16,17,18,19]. Unfortunately, its mechanical sensitivity is far from satisfactory. Thus, accidental stimulus may accelerate the decomposition of CL-20, leading to combustion or explosion. Furthermore, many H and OH free radicals are generated during the decomposition process, and they subsequently release a great deal of heat via a high-speed radical chain reaction. Halogen atoms are known to react with OH or H free radicals and stop the chain reaction, which may reduce the sensitivity of CL-20 [20,21,22]. P-nitrochlorobenzene (PNCB) is an insensitive material and contains halogen atoms, thus it is believed that the combination of CL-20 and PNCB can decrease the sensitivity to a certain degree.

At present, only one or two of the aforementioned technologies have been used to reduce sensitivity in the reported literature. Based upon these research results, stringent sensitivity requirements cannot be met to a certain extent. Thus, we attempted to further reduce the sensitivity of CL-20 by combining the above three methods. Previous work reported that a CL-20/PNA spherical composite with irregular spherical morphology may improve sensitivity [23]. Therefore, we began further exploration into similar composite materials. In this work, a 1:1 molar ratio micro-size spherical composite of PNCB and CL-20 was synthesized via the ultrasound-assisted emulsion (UAE) method. The as-prepared composite displays greatly reduced sensitivity compared to pure CL-20 while retaining high performance due to the incorporation of PNCB. This method requires that the material be handled in a liquid environment in order to guarantee the safety. In addition, the UAE method is a convenient and environmentally friendly approach. This paper presents the first report on reduced sensitivity of the CL-20 composite. Moreover, the morphologies, structures, and properties of the resulting composite were also systematically studied.

## 2. Materials and Methods 

### 2.1. Materials

CL-20 (99.9%) was provided by Qingyang Chemical Industry Corporation (Liaoyang, China) and was used without further purification. P-nitrchlorobenzene (PNCB, 99.5%), ethyl acetate, and gelatin were commercially purchased and used without further purification. Deionized water was prepared with a Milli-Q system (Merck Millipore, Burlington, MA, USA) and used throughout. All the materials were used as received.

### 2.2. Preparation of Micro-Scale CL-20/PNCB Composite

0.53 g CL-20, 0.17 g PNCB and 3 mL ethyl acetate were mixed in a 100 mL beaker at ambient temperature, and the mixture was left to react for about 2 min under vigorous magnetic stirring (25 °C and 1000 r min^−1^). During stirring, 20 mL deionized water and 0.001 g of gelatin (0.2 wt.% of CL-20) were added to obtain an emulsion. 50 mL deionized water was then added to the emulsion, and the mixture was irradiated with ultrasonic waves at 50 kHz. The as-prepared CL-20/PNCB composite was washed several times with deionized water. The as-prepared composite was obtained after evaporation.

### 2.3. Characterization

The reaction was obtained in an SG5200HPT ultrasonic liquid processor (Shanghai Gutel Ultrasonic Instrument Co., Ltd, Shanghai, China), with 30–100 kHz frequency and 30–100 W power. The morphology of the raw materials and the as-prepared composite were examined with a scanning electron microscope (SEM, Ultra-55, Carl Zeiss, Oberkochen, Germany) at 10 kV accelerating voltage for 50 s. The crystal structure of the samples was analyzed using powder X-ray diffraction (PXRD, X’Pert Pro, PANalytical, Almelo, the Netherlands) using Cu-K α radiation (λ = 0.15405 nm) at 40 kV and 40 mA. In the PXRD test, the samples were prepared on silicon single crystal sample holders with 0.1 mm depth. Raman spectra were recorded with a Raman system (inVia, Renishaw, Wotton-under-Edge, UK) using 785 nm laser with about 250 mW power as the excitation source. The spectral resolution of the Raman system was 1 cm^−1^. Fourier-transform infrared (FT-IR) spectra were gathered using a Bruker-Tensor 27 spectrometer (FT-IR, Bruker, Bremen, Germany) with KBr pellets. About 150 mg of KBr was ground in a mortar and pestle, and 1 wt.% of the solid sample was scanned with KBr in a 4000–400 cm^−1^ range at 0.5 cm^−1^ intervals. The FT-IR resolution ratio was 4 cm^−1^. The thermodynamic performance of the samples was analyzed using differential scanning calorimetry (DSC-131, Setaram Instrumentation, Caluire-et-Cuire, France). The samples were placed in an open pan and heated at 10 °C·min^−1^ from 50 to 350 °C in nitrogen steam. The detailed operating conditions are as follows: sample mass ~0.7 mg, heating rate ~10 °C·min^−1^, nitrogen atmosphere (flow rate ~30 mL·min^−1^).

### 2.4. Impact Sensitivity Test

Impact sensitivity was measured using the GJB-772A-97 standard method 601.2 (National Military Standard of China) [24], which has been widely used to measure the H_50_ value. The experimental conditions were as follows: drop weight ~2 kg and sample mass ~30 mg. The impact sensitivity was evaluated by measuring the drop height corresponding to a 50% explosion probability (H_50_).

## 3. Results

### 3.1. CL-20/PNCB Spherical Composite Formation Mechanism

**Figure 1:**
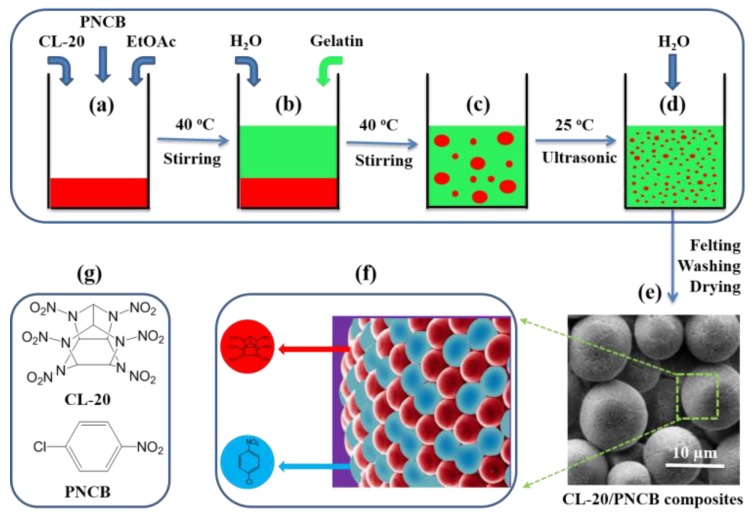
(**a**–**d**) Schematic showing preparation of the spherical CL-20/PNCB composite via the ultrasonic-assisted emulsion (UAE) method. (**e**,**f**) SEM and model images of the target composite. (**g**) Chemical structures of 2,4,6,8,10,12-hexanitro-2,4,6,8,10,12-hexaazaisowurtzitane (CL-20) and P-nitrochlorobenzene (PNCB).

A schematic showing the preparation of the micro-scale CL-20/PNCB composite via the ultrasonic-assisted emulsion (UAE) method is shown in Figure 1. The probable mechanism is as follows. Raw materials including CL-20 and PNCB were dissolved in ethyl acetate (Figure 1a), and then deionized water and gelatin were added into the CL-20/PNCB solution. At this point, phase separation led to the formation of an ethyl acetate-rich bottom layer and a water-rich top layer (Figure 1b). The O/W emulsion system was formed after a surfactant was added with continuous magnetic stirring (Figure 1c). To further reduce the size of the oil phase, the ultrasonic oscillation method was adopted while the homogeneous white emulsion was acquired. The oil phase distributed in the form of spherical droplets in the emulsion due to surface tension. To the best of our knowledge, ethyl acetate has a certain solubility in water. Therefore, ethyl acetate continued to diffuse from the emulsion droplets into the aqueous phase, and adding water accelerated the diffusion process (Figure 1d). When CL-20 and PNCB reached a saturated concentration, the crystal precipitated from the exterior layer to the interior layer. As a result of this rapid process, the composite as-formed not only retained the spherical shape of the oil phase, but also maintained a uniform distribution (Figure 1e,f) [25,26]. In addition, the size of the composite could be controlled by adjusting various parameters, such as solvent ratios, amount of added surfactant, and ultrasonic power [27,28].

### 3.2. Morphology and Structure of the Micro-Scale CL-20/PNCB Composite

**Figure 2:**
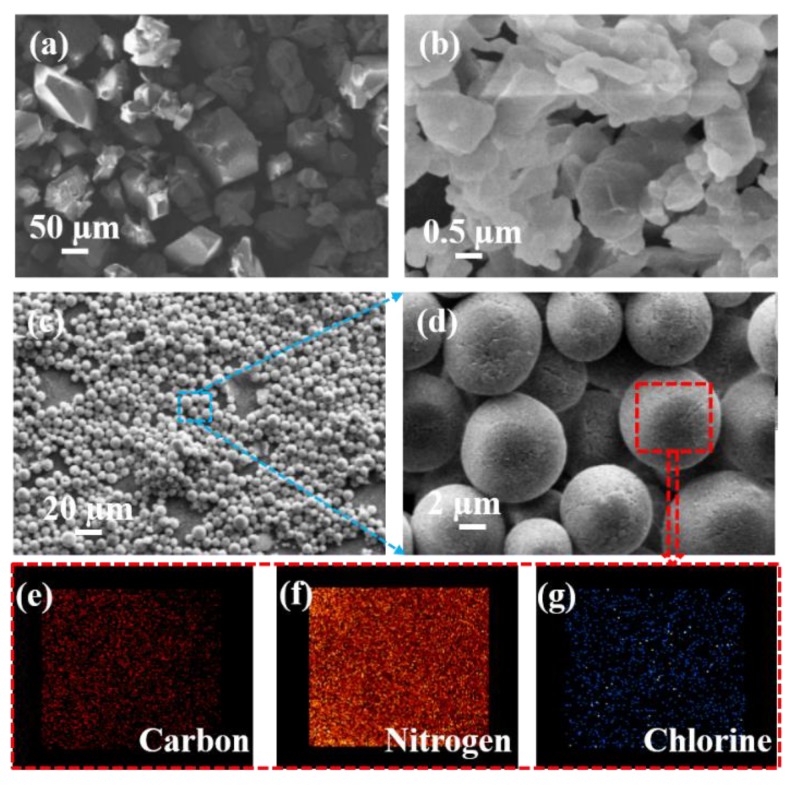
SEM micrographs of (**a**) raw CL-20 crystal, (**b**) raw PNCB crystal, (**c**) and (**d**) micro-sized spherical CL-20/PNCB composite, and (**e**–**g**) the EDX maps of carbon distribution, nitrogen distribution and chlorine distribution, respectively.

Figure 2 shows the morphologies of the raw materials and the as-prepared CL-20/PNCB composite. The raw CL-20 possessed a fusiform microstructure (Figure 2a), and the PNCB was an irregular prismatic crystal (Figure 2b). In contrast, the shape of the CL-20/PNCB composite was spherical with good dispersibility, as shown in Figure 2c. The surface of the composite was smooth, and the average particle size was about 5–8 μm (Figure S1), which was calculated by taking statistics from more than 200 particles, as shown in Figure 2d. The parameters of the CL-20 composite, such as its smooth spherical surface and monodisperse size distribution, presents advantages for reducing its sensitivity.

To study the distribution of the two components in the composite, energy dispersive X-ray spectrometry (EDX) mapping was used to investigate the elemental distribution. The results show that Cl was evenly distributed throughout the spherical composite (Figure 2g), which meant that PNCB was evenly distributed in the composite. At the same time, it was also found that C and N were evenly distributed throughout the spherical composite (Figure 2e,f), which meant that PNCB and CL-20 were evenly distributed in the composite. The above formation of this structure might be caused by H-bonding interactions between NO_2_, Cl, and H in CL-20 and PNCB. Structural characterization methods were analyzed to further investigate the interaction between the two original components.

**Figure 3:**
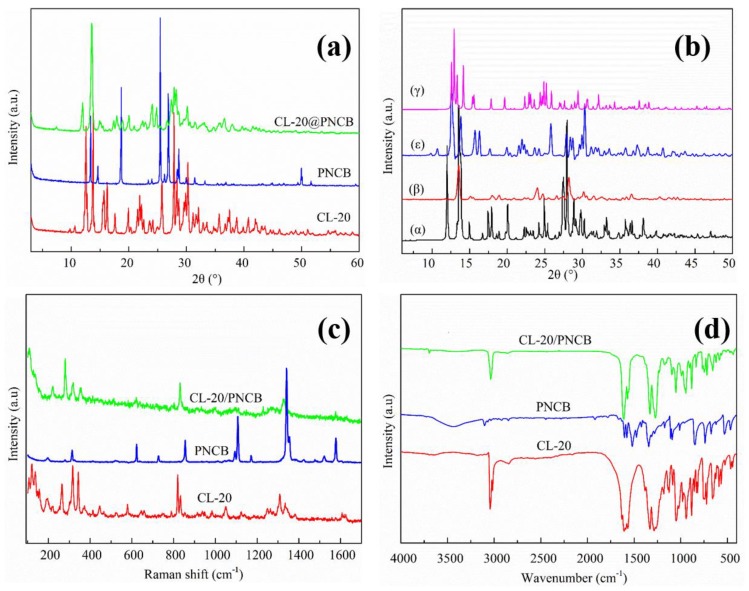
Structural characterization: (**a**) PXRD spectra for raw CL-20, raw PNCB, and the CL-20/PNCB composite. (**b**) PXRD spectra for α-CL-20, β-CL-20, ε-CL-20, and γ-CL-20. (**c**) Raman spectra for raw CL-20, raw PNCB, and the CL-20/PNCB composite. (**d**) FT-IR spectra for raw CL-20, raw PNCB, and the CL-20/PNCB composite.

Powder X-ray diffraction (PXRD) is a well-known characterization method for investigating the formation of a new structure. There are four known crystalline structures of CL-20 (α, β, γ, ε) (Figure 3b) at ambient temperature, which easily lead to the transformation of the crystal type during the synthetic process. Because the initial material CL-20 (Figure 3a) was exactly coincident with pure ε-CL-20 (Figure 3b), one can conclude that the raw CL-20 in the composite was ε-CL-20. The PXRD patterns of the PNCB and as-synthesized samples are also shown in Figure 3a. The peaks localized at 2θ = 11.97°, 13.57°, 24.80°, and 28.21° in the CL-20/PNCB composite were evidently different from those of the initial PNCB and CL-20. The intensity of the diffraction peaks in the CL-20/PNCB composite increased significantly compared with raw CL-20 and PNCB respectively. Some peaks for the CL-20/PNCB composite for 2θ in the 5–50° range disappeared, such as peaks located at 2θ = 12.57°, 21.03°, and 25.77° for CL-20 and 25.37°, 27.14°, and 49.97° for PNCB. New peaks located at 2θ = 14.94° and 28.84° were observed for the composite. These differences enabled easy distinction between the composite and pure PNCB or CL-20. One should also note that this may be due to the formation of a new crystal structure throughout the entire analysis.

Raman spectroscopy is very sensitive towards micro-sized materials and could be used to obtain additional information regarding the micro-sized spherical composite. Raman spectra from CL-20, PNCB and the CL-20/PNCB composite are presented in Figure 3c. CL-20 and PNCB were detected in a grain of crystal from Raman spectroscopy. This illustrates the possible formation of a new structure according to the following irreducible representations. There were several peaks at 265.42, 345.37, 819.75, and 1312.06 cm^−1^ from the CL-20 Raman spectra; these peaks shifted to 280.84, 350.81, 830.73, and 1326.61 cm^−1^ in the composite, respectively, which are ascribed to the C–H bending mode and C–C stretching mode, respectively. Simultaneously, some peak shift also occurred for PNCB. For example, a peak, located at 1354.02 cm^−1^ shifted to 1337.46 cm^−1^, which was attributed to the C–Cl vibration mode. These above-mentioned shifts could be attributed to H-bonding, which changed the symmetry in the spherical composite structure.

FT-IR spectroscopy analyses were carried out in the 4000–400 cm^−1^ range to further determine whether impurities existed in the composite and to understand the interactions between CL-20 and PNCB molecules. FT-IR spectra of raw CL-20, PNCB and the CL-20/PNCB composite are shown in Figure 3d. From a spectroscopy perspective, similar peak locations were observed in the spectrum for the composite and the original materials. For the CL-20/PNCB spherical composite, some absorption due to mixing vibrations in CL-20 occurred at 1043.46, 939.31, 877.59, 586.34, and 557.41 cm^−1^; these peaks intensified and shifted to 1055.03, 941.24, 885.30, 624.92, and 590.20 cm^−1^, respectively. Meanwhile, peaks near 1014.53, 850.59, 734.86, and 669.28 cm^−1^, corresponding to mixing vibrations of PNCB, weakened and shifted to 1055.03, 947.02, 883.37, and 717.50 cm^−1^, respectively. Compared with the original materials, the peaks of the as-prepared CL-20/PNCB composite were located at 447.47, 563.20, 979.81 cm^−1^ (mixing vibration of CL-20), 854.44 cm^−1^ (C–C stretching of CL-20), and 1589.30 cm^−1^ (N=O stretching of CL-20), which also weakened. The peaks at 3101.45 cm^−1^ (C–H stretching of CL-20), 1176.55 cm^−1^ (C–N stretching of PNCB), 1346.24 cm^−1^ (–NO_2_ symmetric stretching of CL-20), and 1525.62 cm^−1^ (–NO_2_ asymmetric stretching of CL-20) were also diminished. The above results agree with the PXRD results and reflected the appearance of a new composite structure.

### 3.3. Thermodynamic Properties and the Sensitivity Performance

**Figure 4:**
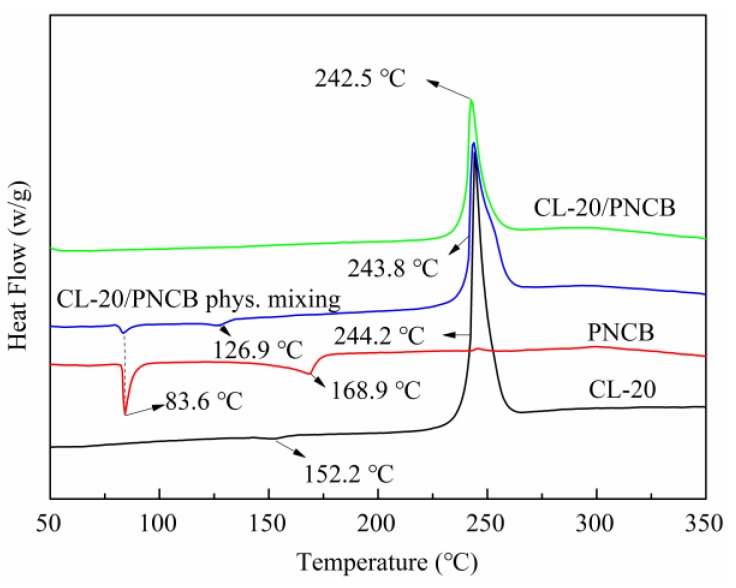
DSC curves for CL-20, PNCB, mixed CL-20/PNCB, and the CL-20/PNCB composite.

The thermal behavior of the composite was examined using DSC. The DSC curves in Figure 4 show that the as-prepared spherical composite and the initial materials exhibit different thermodynamic behavior. Pure PNCB exhibited two endothermic peaks at 83.6 and 168.9 °C. The change in conformation at 152.2 °C (ε → γ phase) and the decomposition peak of CL-20 (244.2 °C) were observed in the curve for pure CL-20. The DSC curve of the physical mixture was very similar to that of the two initial materials. However, no phase change was observed in the CL-20/PNCB composite and exhibited a single sharp melting exothermic peak at 242.5 °C. In the as-prepared CL-20/PNCB composite, there might be a new chemical structure formed by the hydrogen bond between C–H and nitro groups in CL-20 molecules and the C–H, Cl–, and nitro groups in PNCB molecules, which resulted in fracture of the chemical bonds in CL-20 or PNCB in the as-prepared composite. Furthermore, the decomposition temperatures were different. 

The impact sensitivity was measured to evaluate the safety of the CL-20/PNCB composite, and the results are shown in Table 1. The drop height, corresponding to a 50% explosion probability for the as-formed composite, was significantly higher than those of the physical mixture (31 cm) and the original material CL-20 (13 cm). In particular, the drop height of CL-20/PNCB composite ranged up to 63 cm, which is a significant enhancement compared with previously reported values for CL-20/DNB [12] and CL-20/TNT [17] composites (Table 1). We also built tables comparing the explosive properties (e.g., DSC, Sensitivity, Velocity of detonation (VOD), and Detonation pressure (DP)) of known composites with CL-20 (Table S1).

The sensitivity closely relates to the structure, shape, and size of the materials. The newly formed structure and the interaction between the molecules in the as-prepared composite could increase the stability of the crystal structure and contribute to reduced sensitivity. Similarly, a CL-20/PNCB composite with extremely small size also exhibited size-dependent properties, including thermal decomposition, operational performance, and especially sensitivity. It was interred that the average diameter of small particles was too small to become a hot spot during impact stimulus, thus leading to decreased impact sensitivity. Meanwhile, facing external stimuli, the CL-20/PNCB spherical composite could dramatically improve insensitivity over non-spherical crystals by reducing friction among neighboring particles. For the foregoing reasons, the impact sensitivity of the as-prepared CL-20/PNCB spherical composite in this paper exhibited satisfactory results. This work on new energetic micro-size spherical composites opens up new avenues for research into high EMs. 

### 3.4. Detonation Performance

The important step for the development of new energetic composite is to accurately predict their detonation properties, including detonation velocity (D) and detonation pressure (P), which often play an important role in potential applications. The detonation pressure of the CL-20/PNCB composite may be calculated with a simple empirical equation: P = ${\mathrm{Kp}}_{o}^{2}$ψ, K = 15.58, ψ = NM^1/2^Q^1/2^. Detonation velocities can be calculated with the equation D = Aψ^1/2^(1 + Bp_o_), where A = 1.01 and B = 1.30. N is the number of moles of gaseous detonation products per gram of explosive, M is the average weight of these gases, Q is the chemical energy of the detonation reaction, and p_o_ is the initial density. Values of N, M, and Q may be estimated under the assumption of arbitrary decomposition [29]. The results indicate that the detonation velocity (7494 m/s) and detonation pressure (25.5 GPa) of the composite are slightly reduced relative to CL-20. 

## 4. Conclusions

Herein, we facilely fabricated a monodisperse, micro-scale spherical energetic composite composed of CL-20 and PNCB via the UAE method. UAE is a convenient, environmentally friendly, and rapid process. The results indicate that the CL-20/PNCB composite showed better thermodynamic stability. The drop height corresponding to a 50% explosion probability revealed that the micro-scale CL-20/PNCB spherical composite was more insensitive compared with other CL-20-based EMs composites. In addition, the current work could provide a detailed understanding into the preparation of insensitive EMs. We anticipate that this approach will facilitate further design of insensitive EMs.

## Figures and Tables

**Table 1:** Impact sensitivity of CL-20 based composite.

CL-20 Based Composite or Co-Crystal	Impact Sensitivity (H_50_/cm)	Shape	Particle Size (μm)
CL-20/DNB [12]	55	polyhedron	300
CL-20/TNT [18]	30	prism	500
As-prepared CL-20/PNCB	63	sphere	5–8
CL-20/PNCB mixture	31	polyhedron	2–10

## Supplementary Materials

The following are available online at http://www.mdpi.com/1996-1944/11/7/1130/s1, Figure S1: Particle size distribution of as-formed CL-20/PNCB composite, Table S1: Make comparable with every explosive properties (Eg:Sensitivity, VOD and DP, etc.) with already known composites with CL-20.
