# Supplementary material for: Synthesis, Characterization, and Sensitivity of a CL-20/PNCB Spherical Composite for Security
Source: Materials (Basel). 2018 Jul 3;11(7):1130. doi: 10.3390/ma11071130 (PMC6073270; doi:10.3390/ma11071130)
Supplement: Supplementary file 1 [file materials-11-01130-s001.pdf]

Supplementary Materials

Synthesis, Characterization, and Sensitivity of a CL-20/PNCB Spherical Composite for Security

Yanfang Zhu,[1] Yuewen Lu,[1] Bing Gao,[1] Dunju Wang,[1] Changping Guo*[1] & Guangcheng Yang[2]

[1] Sichuan Co-Innovation Center for New energetic materials, Southwest University of Science and Technology (SWUST), Mianyang 621010, China

[2] Institute of Chemical Materials, China Academy of Engineering Physics (CAEP), Mianyang 621900, China

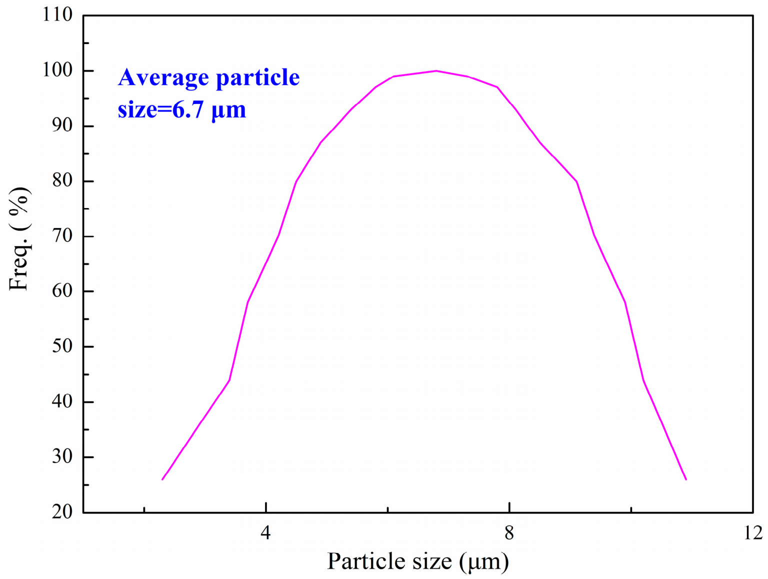

Figure S1. Particle size distribution of as-formed CL-20/PNCB composite.

We also make a comparable table with all explosive properties (e.g, DSC, sensitivity, VOD and DP, etc.) of already known composites with CL-20.

Table S1. Impact sensitivity, detonation velocity and detonation pressure of CL-20 based composite.

CL-20 Based Composite or Co-Crystal	Impact Sensitivity	Detonation Velocity (m/s)	Detonation Pressure (GPa)
CL-20/DNB [1]	55 cm	8434	34.0
CL-20/TNT [2]	30 cm	8426	32.3
CL-20/TATB [3]	/	9127	41.3
CL-20/BTF [4]	/	8969	39.1
As-prepared CL-20/PNCB	63cm		

Thermal analysis:

CL-20/DNB：There are two exothermic peaks at 216.8 ℃ and 242.8 ℃, respectively.

CL-20/TNT：In the DSC curve, the co-crystal has a narrow endothermic peak with onset at 134℃, which is denoted as the melting point of the co-crystal. Two obvious and rapid weight losses occur in the temperature ranges of 214–221 ℃ and 238–249 ℃ for the co-crystal, respectively.

CL-20/TATB: The peak at 208.15 ℃ may demonstrate the phase transition temperature of the co–crystal. The DSC profile curve reveals a strong exothermic peak at 231.8 ℃.

CL-20/BTF：The DSC profile in the DSC curve reveals a strong exothermic peak at 235 °C, attributed to the decomposing event of the cocrystal.

References

1. Wang, Y.P., Yang, Z.W., Li, H.Z., Wang, J.H., Zhou, X.Q., Zhang, Q. A novel cocrystal explosive of HNIW with good comprehensive properties. *Propellants, Explos., Pyrotech.* **2014**, *39*, 590–596.
2. Yang, Z.W, Li, H.Z, Huang, H, Zhou, X.Q, Li, J.S, Nie, F.D. Preparation and performance of a HNIW/TNT cocrystal explosive. *Propellants, Explos., Pyrotech.* **2013**, *38*, 495–501.
3. Xu, H.F, Duan, X.H, Li, H.Z, Pei, C.H. A novel high-energetic and good-sensitive cocrystal composed of CL-20 and TATB by a rapid solvent/non-solvent method. *RSC Adv.* **2015**, *5*, 95764–95770.
4. Yang, Z.W, Li H.Z, Zhou X.Q, Zhang C.Y, Huang H, Li J.S, Nie F.D. Characterization and Properties of a Novel Energetic−Energetic Cocrystal Explosive Composed of HNIW and BTF. *Cryst. Growth Des*. **2012**, *12*, 5155−5158.
